# A data-driven approach to optimising the encoding for multi-shell diffusion MRI with application to neonatal imaging

**DOI:** 10.1002/nbm.4348

**Published:** 2020-07-06

**Authors:** Jacques-Donald Tournier, Daan Christiaens, Jana Hutter, Anthony N. Price, Lucilio Cordero-Grande, Emer Hughes, Matteo Bastiani, Stamatios N. Sotiropoulos, Stephen M. Smith, Daniel Rueckert, Serena J. Counsell, A. David Edwards, Joseph V. Hajnal

**Affiliations:** 1 Department of Biomedical Engineering, School of Biomedical Engineering and Imaging Sciences, King's College London, King's Health Partners, St. Thomas' Hospital, London, UK; 2 Centre for the Developing Brain, School of Biomedical Engineering and Imaging Sciences, King's College London, King's Health Partners, St. Thomas' Hospital, London, UK; 3 Wellcome Centre for Integrative Neuroimaging - Oxford Centre for Functional Magnetic Resonance Imaging of the Brain (FMRIB), University of Oxford, Oxford, UK; 4 Sir Peter Mansfield Imaging Centre, School of Medicine, University of Nottingham, Nottingham, UK; 5 Biomedical Image Analysis Group, Imperial College London, London, UK

**Keywords:** diffusion MRI, HARDI, multi-shell, neonatal imaging

## Abstract

Diffusion MRI has the potential to provide important information about the connectivity and microstructure of the human brain during normal and abnormal development, noninvasively and in vivo. Recent developments in MRI hardware and reconstruction methods now permit the acquisition of large amounts of data within relatively short scan times. This makes it possible to acquire more informative multi-shell data, with diffusion sensitisation applied along many directions over multiple *b*-value shells. Such schemes are characterised by the number of shells acquired, and the specific *b*-value and number of directions sampled for each shell. However, there is currently no clear consensus as to how to optimise these parameters. In this work, we propose a means of optimising multi-shell acquisition schemes by estimating the information content of the diffusion MRI signal, and optimising the acquisition parameters for sensitivity to the observed effects, in a manner agnostic to any particular diffusion analysis method that might subsequently be applied to the data. This method was used to design the acquisition scheme for the neonatal diffusion MRI sequence used in the developing Human Connectome Project (dHCP), which aims to acquire high quality data and make it freely available to the research community. The final protocol selected by the algorithm, and currently in use within the dHCP, consists of 20 *b=0* images and diffusion-weighted images at *b* = 400, 1000 and 2600 s/mm^2^ with 64, 88 and 128 directions per shell, respectively.

## 1 Introduction

Diffusion MRI (dMRI) has been the focus of intense research over the last 20 years, holding great promise for investigation of tissue microstructure due to the technique's unique sensitivity to the micron-scale diffusion of water. In recent years, there has been increasing interest in the use of so-called multi-shell dMRI, driven to a large extent by improvements in image acquisition and reconstruction methods, which can now provide the amount of data required within clinically feasible scan times. A number of promising methods have been proposed to model data of this nature (eg, Jensen et al^1^; Jeurissen et al^2^; and Kaden et al, among others^3–6^). Many of these approaches are based on the so-called standard model of diffusion,^7^ consisting of intra- and extra-axonal compartments convolved with a corresponding fibre orientation function, optionally with an additional isotropic cerebro-spinal fluid compartment.^6,8–16^

More recent work has also investigated the use of additional contrast mechanisms, such as spherical and planar tensor encoding,^17–20^ more complex Q-space trajectories,^21^ variable echo times^22^ and variable inversion times,^23,24^ among others. Many of these entail a considerable increase in scan time, and the use of custom sequences, limiting their widespread use in the immediate future. Furthermore, these methods are still under active research, and a consensus has yet to be reached regarding which of these should be deployed. We therefore decided to focus on optimisation of the multi-shell pulsed gradient spin echo (PGSE) sequence, as it is likely to remain the most widely used acquisition strategy for the foreseeable future, especially for large longitudinal and cross-sectional studies.

**Figure 1:**
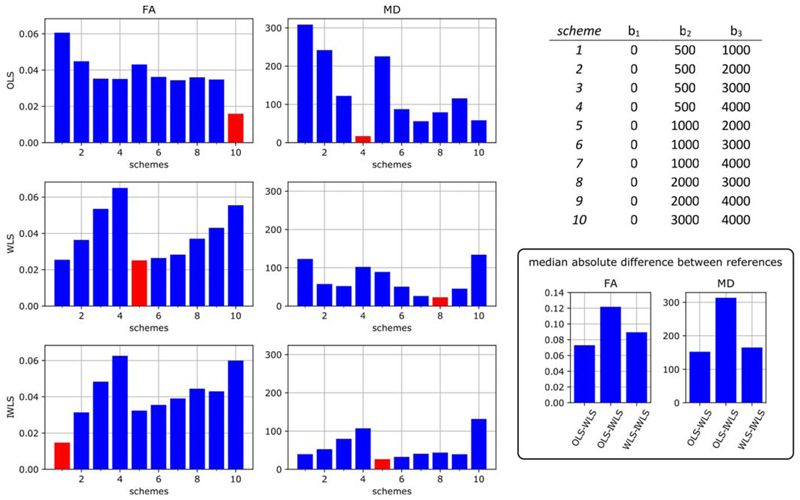
Optimising sampling schemes for measuring fractional anisotropy (FA) and mean diffusivity (MD) using minimal discrepancy relative to a superset of all available data for three different DTI analysis methods (ordinary least squares [OLS], weighted least squares [WLS] and iteratively reweighted least squares [IWLS]). The optimal protocols, which are different in each case, are highlighted in red. The bar graphs show the median absolute difference in DTI metrics obtained using all the data compared with those obtained using different subsets of three of the available *b*-values. The results for MD are shown in units of μm^2^/s (FA is dimensionless). Differences were computed voxel-wise, and the median absolute difference was calculated over the whole brain for each of the five subjects included in the study, then averaged across subjects. Results are shown for FA (left) and MD (centre), computed using three commonly used estimators: OLS (top row), WLS (middle row) and IWLS (bottom row). The x-axis corresponds to the numbered schemes shown in the table on the top right, which specify the included shells. The schemes with the lowest discrepancy are highlighted in red. The bottom right panel shows the equivalent median absolute difference between the metrics obtained with the different fitting approaches using all the available data (ie, the difference between the references used for the other plots)

A common requirement for researchers when setting up a multi-shell dMRI protocol is the determination of optimal imaging parameters, particularly the number of *b*-value shells and the number of diffusion-weighting directions and the *b*-value per shell. However, there are many different reconstruction algorithms available, and no clear consensus as to which is best. The lack of ground truth is also problematic, motivating the use of hardware phantoms with known fibre configurations.^25^ This makes it very difficult to identify optimal imaging parameters, since any optimisation will typically aim to reduce errors in the estimated parameters of a given model; this obviously does not provide any guarantees that the data would be suitable for a different (possibly yet to be devised) reconstruction algorithm. To illustrate the difficulties with such an approach, Figure shows the median absolute deviation in DTI metrics computed using different subsets of an extended data acquisition to seek protocols that produce low errors. The approach is to calculate mean diffusivity (MD) and fractional anisotropy (FA) using a target analysis method of choice for all the available data (six shells, 50 directions on each, five subjects) to determine a “reference value” and then to do the same using only three of the shells to find feasible duration protocols that are reliable in the sense of producing the lowest median error (see the Appendix for full details). This was performed using three different commonly used tensor estimation strategies, namely, ordinary least-squares (OLS), weighted least-squares (WLS) and iteratively reweighted least-squares (IWLS),^26–29^ all implemented within *MRtrix3*. Depending on the analysis method used, different “optimal” protocols are identified (see the Figure 1 caption for details). It is striking that this is the case even although the same model of diffusion (the diffusion tensor) is assumed for all cases.

There is therefore a need for a means to determine acquisition parameters that provide the most eloquent, information-rich data in a manner agnostic to any particular reconstruction algorithm. This is particularly important for large-scale data collection projects that will provide a shared resource to be used by diverse academic groups for purposes that the users, rather than the data collectors, define.

This study was motivated by the need to optimise the dMRI acquisition scheme for the developing Human Connectome Project (dHCP), which aims to acquire structural, functional and diffusion MRI data from more than 1000 neonates and fetuses, among other clinical, behavioural and genetic measures (http://www.developingconnectome.org/). Since these data are to be made freely available to the wider neuroimaging community, it was important to acquire data suitable for the widest possible range of analysis methods. Moreover, the diffusion-weighted (DW) signal in the perinatal age range differs markedly from that in adults, in that it exhibits much higher apparent diffusion (suggesting increased extracellular and reduced intracellular content), and much lower anisotropy. Furthermore, these characteristics vary strongly as a function of both location and age, as the various structures in the brain mature at different rates. Much analysis work to date has used algorithms designed for adult studies, but there is clearly scope for optimising all aspects of the analysis/modelling pipeline and this could result in methods that are different from existing approaches.

Taken together, these factors provide strong motivation to develop a data-driven strategy that can be used to design data acquisitions for a desired target subject group, optimised for information content rather than to support a predefined processing method. To address this challenge, we propose to use an information-theoretic approach, similar in spirit to our previous work on optimising single-shell dMRI parameters.^30^

## 2 Methods

In this paper, we propose a framework to determine the optimal number of shells, along with the corresponding *b*-value and the number of directions sampled. To make this problem tractable, we separate the *b*-value dependence from the orientation dependence and treat each problem separately. For the *b*-value dependence, the central concept is then to identify a linear basis to represent the *b*-value dependence of the data (voxel values) as measured empirically, and to determine the number of coefficients of that basis that can realistically be measured in practice. Given this number, the task is then to identify a set of parameters (i.e., the number of shells, *b*-value per shell and the number of DW volumes per shell) that provide optimal sensitivity to these coefficients.

The angular dependence was itself the focus of previous work, applied to the adult case^30^; here, we simply deploy the same methodology to determine the per-shell sampling density required to capture the detectable number of spherical harmonics. Briefly, the approach expresses the dMRI signal for a single *b*-value shell using the spherical harmonics basis, and identifies the highest angular frequency term that can realistically be detected in the data. This analysis is performed only in voxels deemed to contain a single-fibre population, since these contain the highest angular frequency content, and also because this allows averaging across voxels (after realignment of the fibre direction to a common axis). For further details, the reader is referred to Tournier et al.^30^ This analysis provides an estimate of the angular frequency content at each *b*-value, which translates directly into the corresponding minimum number of directions required for nonlossy sampling (i.e., without aliasing in the angular domain). Once the optimal *b*-values have been identified, this analysis can be used to ensure that the sampling density within each shell is adequate.

To determine the sampling requirements across *b*-values, we focus on the *b*-value dependence of the orientationally averaged raw DW signal as a function of *b*-value. Assuming sufficiently dense and uniform sampling, the mean DW signal over a single *b*-value shell is rotationally invariant, and independent of the fibre arrangements.^3,31,32^ Near-uniform distribution of gradient directions can be obtained using electrostatic repulsion approaches,^33,34^ as used in this study, and sufficient sampling density can be verified using the approach described above.^30^

As an overview, this study consists of the following steps:

- Acquire data that are sufficiently densely sampled in the *b*-value and angular domains to capture all the expected features of the DW signal.
- Identify a suitable data-driven linear basis to represent the signal.
- Estimate the effect size observed in the signal for each coefficient of that basis.
- Identify the set of acquisition parameters that provide optimal sensitivity to these coefficients.

The optimisation of the acquisition parameters (the last step in the list above) was performed by:

- For a given set of *b*-values and corresponding number of directions, define a measure of sensitivity to the coefficients of interest.
- For a given set of *b*-values, optimise this sensitivity measure with respect to the number of directions per *b*-value shell.
- Given a fixed number of *b*-value shells, optimise the sensitivity measure with respect to the value of these *b*-values (with the corresponding number of directions optimised as per the previous step).

Each of these steps is described in more detail in the following sections.

### 2.1 Data acquisition and preprocessing

Data were acquired from five neonates scanned at term-equivalent age (see Table 1 for details) on a Philips Achieva 3 T magnet at the Evelina Newborn Imaging Center at St. Thomas’ Hospital (London, UK). The study was approved by the National Research Ethics Committee and written informed parental consent was obtained prior to scanning. The system was equipped with a standard 32-channel head coil, using a DW pulsed gradient spin-echo EPI sequence (TE/TR = 70/2260 ms, 2 × 2 mm voxel size, 11 slices with 2 mm thickness with a 1 mm slice gap, 112 × 112 matrix, SENSE factor 2, single anterior–posterior phase-encode direction). Data were collected along 50 noncollinear directions optimised using electrostatic repulsion^33^ at *b*-values of 500, 1000, 2000, 3000 and 4000 s/mm^2^, along with five *b=0* s/mm^2^ volumes. These directions were optimised using the *MRtrix3* “dirgen” command, and the same set of 50 directions was used for each shell. We note that in adults, a spherical harmonic order of 8 (45 parameters) was shown to be sufficient to capture all realistically measurable components of the signal, even up to *b* = 5000 s/mm^2^.^30^ Therefore, the 50 directions used in the acquisition here are sufficient to satisfy this criterion, especially considering that neonatal data will exhibit lower anisotropy than observed in adults.^35–37^

**Figure 2:**
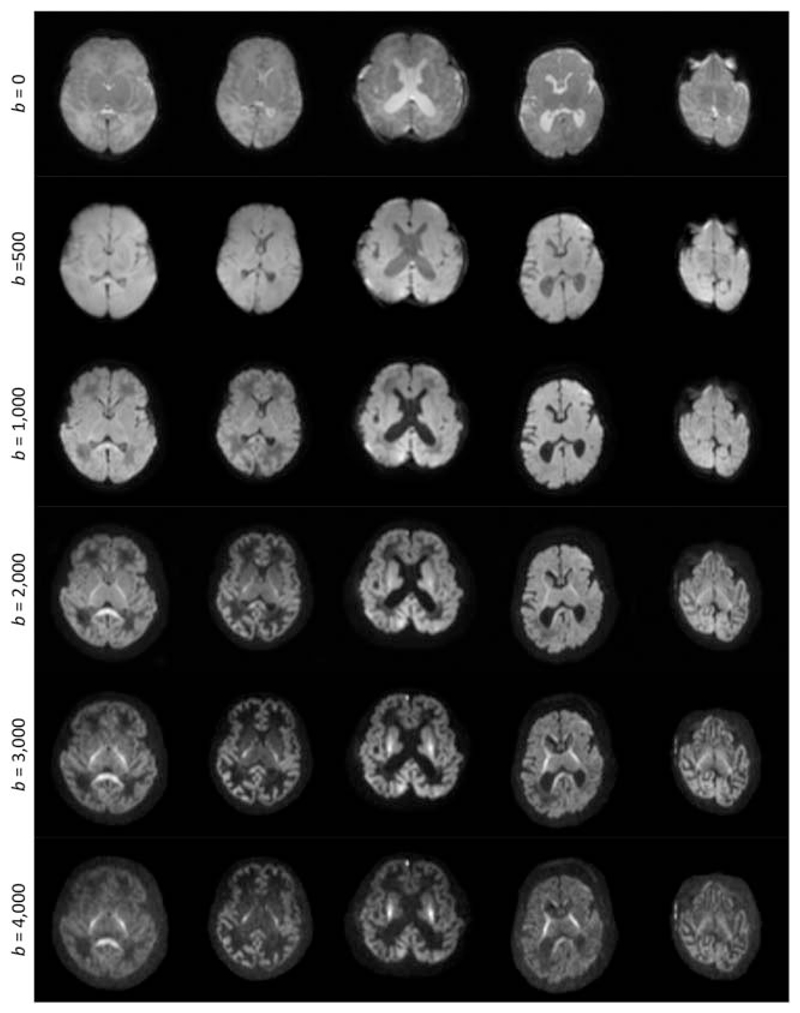
Images of the mean raw dMRI signal for all five subjects (columns), for each *b*-value (rows). These constitute the input for the optimisation algorithm. Note that the images are windowed independently for each *b*-value to allow visualisation of the contrast in the high *b*-value images

For each subject, the data were first corrected for motion- and eddy current-induced distortions using the FSL EDDY (version 5.0.8).^38,39^ The mean DW signal for each shell was then computed within a brain mask. Typical images of the resulting mean signal are shown in Figure 2.

### 2.2 Identifying a suitable linear basis

In contrast to the angular domain, where spherical harmonics form a natural basis,^30^ there is no such natural basis to represent the *b*-value dependence of the signal. While some bases have been proposed,^40–47^ there is an inherent scale dependence that makes the analysis dependent on user-defined parameters (*D*).^48^ For this reason, we adopted a fully data-driven, model-free basis, derived using matrix decomposition approaches.

We use the compact singular value decomposition (SVD), which decomposes a general *m* × *n* matrix *A* (with *m* ≤ *n*) as *USV^T^*, where *U* is a *m* × *m* orthonormal matrix, *S* is a *m* × *m* diagonal matrix of singular values, and *V^T^* is a *m* × *n* orthonormal matrix. To make use of this decomposition, we form the compact SVD of the transpose of *D*:

(1) $$D=\text{U}SV^{T}.$$

More specifically, we consider a multi-shell protocol consisting of *N_s_* *b*-values, with *n_s_* uniformly distributed DW directions for the shell index *s*. We form the *N_s_* × *N_v_* matrix *D* consisting of the mean DW signal for each brain voxel at each *b*-value, where *N_v_* is the number of voxels in the subject-specific brain mask. We wish to decompose *D* into a *N_s_* × *N_v_* matrix *W* of per-voxel weights, and a *N_s_* × *N_s_* orthonormal basis matrix *H*, such that:

(2) D=HW.

The weight and basis matrices can then be obtained as:

(3) $$\begin{array}{l} H=\text{U}\\ W=\text{S}{\text{V}}^{T} \end{array}$$

Furthermore, thanks to the properties of the SVD, the singular values (i.e., the diagonal of *S*) can be used to obtain an estimate of the typical component-wise effect sizes *ϵ* (this evaluates to the root-mean-square value of the weights across the rows of *W*):

(4) $$\epsilon =\frac{1}{\sqrt{N_{v}}}\text{diag}\left(S\right).$$

### 2.3 Measure of sensitivity

The decomposition described above provides a linear breakdown of the effects observed in the data, and of their relative contributions. This can be used to predict the sensitivity to these various effects for a candidate acquisition with the same *b*-value shells, but different numbers of volumes per shell. Assuming independent and identically distributed Gaussian noise, the expected variance in the measured mean DW signal for each shell *s* is simply $\sigma_{s}^{2}=\sigma^{2}/n_{s}$, with *n_s_* the number of volumes in shell *s*, and *σ* ^2^ the variance per measurement in the data. This rests on the observation that provided the sampling density is uniform and sufficiently dense (as per Tournier et al^30^), the variance in the mean DW signal for a given shell is inversely proportional to the number of DW volumes acquired at that *b*-value; the mean DW signal is orientationally invariant, and each volume contributes equally to its estimation.

The predicted variance in the mean per-shell signal can be used to predict the variance in the estimated basis coefficients using the law of propagation of errors.^49^ Based on Equation 1, if *d* = *Hw* is the vector of mean per-shell signals for a single voxel (where *w* is the corresponding vector of basis coefficients), the variance–covariance matrix Σ_*w*_ for the coefficients *w* is related to the corresponding variance–covariance matrix Σ_*d*_ for the mean per-shell signals *d* according to:

(5) $$\Sigma_{d}=H\Sigma_{w}H^{T}.$$

Since *H* is orthogonal by construction, *H* ^−1^ = *H^T^*, yielding:

(6) $$\Sigma_{w}=H^{T}\Sigma_{d}H.$$

With repeated measurements per shell, $\Sigma_{d}=\text{diag}\left(\sigma_{s}^{2}\right)=\text{diag}\left(\sigma^{2}/n_{s}\right)$ (see above). Hence:

(7) $$\Sigma_{w}=\sigma^{2}H^{T}\text{diag}\left(\frac{1}{n_{s}}\right)H.$$

Finally, we compute the coefficient of variation (CV) for coefficient *c* as the ratio of its standard deviation (given by the square root of its variance Σ*_w_*(*c*,*c*)) to its effect size *ϵ_c_* (as provided in Equation 4):

(8) $$\text{C}{\text{V}}_{c}=\frac{\sigma_{c}}{\epsilon_{c}}=\frac{\sqrt{\Sigma_{w}\left(c,c\right)}}{\epsilon_{c}}\;\text{with}\;\Sigma_{w}\left(c,c\right)=\sigma^{2}\sum_{s}^{N_{s}}\frac{H_{sc}^{2}}{n_{s}}.$$

Note that CV*_c_* is equivalent to the inverse of the contrast-to-noise ratio (CNR) of the coefficient of interest.

### 2.4 Optimising the number of directions per shell

The optimal acquisition should strive to minimise the combined CV of the *N_c_* estimated coefficients. We therefore use the sum of squared coefficients of variation (SSCV) as a measure of optimality:

(9) $$\text{SSCV}=\sum_{c}^{N_{c}}{\text{CV}}_{c}^{2}=\sum_{c}^{N_{c}}\frac{\sum_{w}\left(c,c\right)}{\in_{c}^{2}}=\sigma^{2}\sum_{c}^{N_{c}}\left[\frac{1}{\in_{c}^{2}}\sum_{S}^{N_{s}}\frac{H_{SC}^{2}}{n_{s}}\right].$$

Note that if the effect size were the same for all coefficients, this is proportional to the sum of the variances, which is appropriate for independent measurements. However, when effect sizes differ, this measure is most strongly influenced by the coefficients with the highest CV, pushing the optimisation towards parameters that maximise the CNR of the noisiest components, without overly penalising high CNR components.

We now need to derive the number of directions per shell ***n*** = {*n_s_*} that maximise the sensitivity, and hence minimise the SSCV, within the constraint that the number of DW volumes, *N*_total_, is fixed by scan time limitations:

(10) $${\text{n}}_{\text{opt}}=\underset{\text{n}}{\text{min}}\;\text{SSCV}\;\text{such}\;\text{that}\sum_{S}^{N_{s}}n_{s}=N_{\text{total}}.$$

This can be solved using Lagrange multipliers:

(11) $$ℒ(\text{n},\lambda )=\text{SSCV}+\lambda \sum_{s}^{N_{s}}n_{s}=\sigma^{2}\sum_{c}^{N_{c}}\left[\frac{1}{\epsilon_{c}^{2}}\sum_{s}^{N_{s}}\frac{H_{sc}^{2}}{n_{s}}\right]+\lambda \sum_{s}^{N_{s}}n_{s}.$$

The derivative with respect to the number of directions in shell *s* is:

(12) $$\frac{ℒ(n,\lambda )}{n_{s}}=-\sigma^{2}\sum_{c}^{N_{c}}\frac{H_{sc}^{2}}{\epsilon_{c}^{2}n_{s}^{2}}+\lambda =-\frac{\sigma^{2}}{n_{s}^{2}}{\sum_{c}^{N_{c}}\left(\frac{H_{sc}}{\epsilon_{c}}\right)}^{2}+\lambda .$$

Setting the derivative to zero yields:

(13) $$n_{s}^{2}=\frac{\sigma^{2}}{\lambda }{\sum_{c}^{N_{c}}\left(\frac{H_{sc}}{\epsilon_{c}}\right)}^{2}.$$

This therefore provides the optimal number of DW volumes for shell *s* relative to the other shells. Since we assume that the total number of volumes is fixed by scan time constraints, the absolute numbers can trivially be obtained by normalising to *N*_total_:

(14) $$n_{s}=n_{s}^{*}N_{\text{total}}/\sum_{s}^{N_{s}}n_{s}^{*},$$

where $n_{s}^{*}$ is the value obtained by setting *λ* in Equation 13 to an arbitrary constant. It is interesting to note that with this derivation, the fractions of measurements on each shell are independent of *N*_total_ or *σ*: they depend purely on the intrinsic effects in the DW signal.

### 2.5 Optimising the *b*-values

So far, the derivation above holds for data acquired over a fixed number of shells with predetermined *b*-values. If a sufficiently large number of different combinations of shells and *b*-values were available, this could be used to identify the optimal set of parameters, by searching for the subset of *b*-values with minimum SSCV (Equation 9). It is, however, impractical to acquire data over a sufficient number of *b*-values to ensure the search is exhaustive, particularly in a neonatal cohort where scan times must be kept short.

We therefore used interpolation methods to resample the acquired data, allowing the generation of predicted data for any combination of *b*-values; this approximation is justified on the grounds that the diffusion signal varies smoothly as a function of *b*-value, so that interpolation errors are likely to be minimal. For this purpose, we use the Piecewise cubic Hermite Interpolating Polynomial (PCHIP) algorithm^50^ available in Matlab (MathWorks, Natick, MA, USA), which has the desirable property of explicitly preserving monotonic behaviour between sample points.

Given a fixed desired number of shells, the optimal *b*-values for each shell are determined using a simple nonlinear optimisation approach (the Nelder–Mead simplex algorithm,^51^ as implemented in Matlab’s *fminsearch* routine). For each iteration, the mean DW signals per voxel at the current *b*-values are obtained by PCHIP interpolation from the measured data. The SSCV of the parameters is then optimised with respect to the number of directions per shell as described earlier in Optimising the number of directions per shell (section 2.4). Finally, the optimised SSCV value is used as the cost function for the search over *b*-values.

### 2.6 Accounting for *T*_2_ decay

So far, the derivation has ignored the effect of *T*_2_ decay on the sensitivity of the acquisition. In practice, the shell with the highest *b*-value will dictate the echo time of the entire acquisition (assuming a constant echo time is desired, as is typically the case), and this will have a direct impact on the SNR of the data. To account for these effects, we include a *b*_max_-dependent *T*_2_ decay term, assuming a standard PGSE sequence, similar to Alexander and Barker,^52^ with square diffusion-sensitising gradients and the following parameters:

- A 5 ms delay between the 90° excitation pulse and the onset of the first DW gradient pulse.
- A 5 ms pulse duration for the 180° refocusing pulse (including slice selection gradients and crushers, if any).
- A 15 ms delay between the end of the second DW gradient pulse and echo time.
- 80 mT/m gradient strength.

The attenuation factor is then computed as exp(−*TE*/*T*_2_), and is used to scale the effect sizes in Equation 4, inherently penalising parameters sets that include a high *b*-value shell. Results were generated assuming *T*_2_ = 100, 150 and 200 ms, which are typical for neonatal brain,^53^ as well as ignoring *T*_2_ relaxation.

### 2.7 Analysis within and across subjects

As described, the analysis requires input data in the form of a *N_s_* × *N_v_* matrix corresponding to the mean DW signal for each of the *N_s_* = 6 *b*-value shells across all *N_v_* brain voxels. The analysis was applied for each subject independently within a mask of the brain voxels (with *N_v_* ranging from 14 894 to 23 481 voxels).

In addition, the analysis was also performed on a single dataset formed by concatenating the data for all subjects along the slice axis, for a combined total of *N_v_* = 99 306 voxels. Unless otherwise stated, the results shown were produced with this combined analysis.

## 3 Results

**Figure 3:**
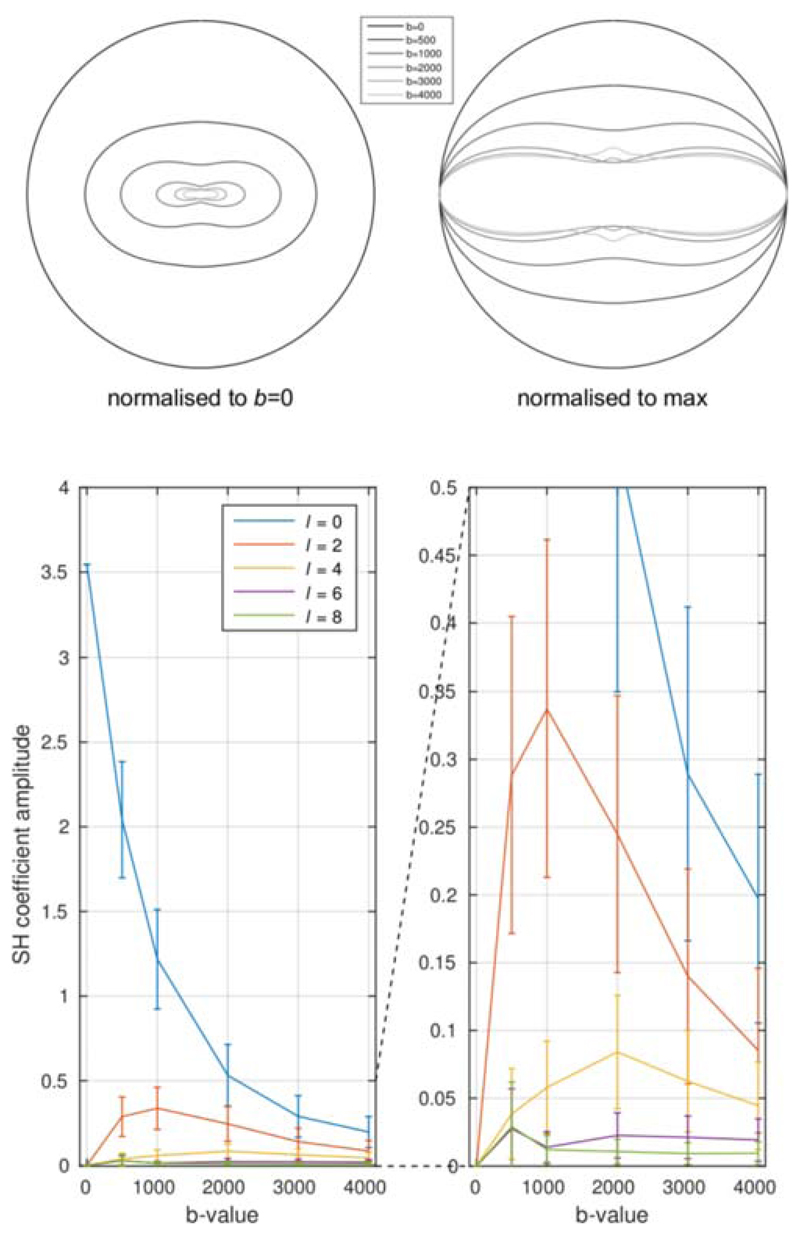
Angular frequency content of dMRI signal in neonates, as a function of *b*-value (cf.^30^). Top: polar plot of the signal (fibre orientation is along the vertical axis), showing the rapid decrease in signal with increasing *b*-value, along with the expected increase in angular contrast. Bottom: a plot of the corresponding spherical harmonic coefficient amplitudes. Angular frequencies above l = 4 are already within the noise floor, although there is a suggestion that l = 6 might be detectable for *b* ≥ 2000 s/mm^2^. This implies that a minimum of 15 and 28 DW directions are required for *b* < 2000 s/mm^2^ and *b* ≥ 2000 s/mm^2^, respectively

The angular domain analysis shows that the angular frequency content is much lower in neonates (Figure 3) than has been shown previously in adults,^30^ as expected. In our data, spherical harmonic terms of order 8 and above are indistinguishable from noise for any realistic SNR value (Figure S1). Note that this does not imply that these terms are strictly zero, but that they can, for all intents and purposes, be neglected for any reasonable voxel-wise analysis. Order 6 terms were also small but could be observed with *b* ≥ 2000 s/mm^2^, implying that a minimum of 28 directions are required at these *b*-values. At lower *b*-values, only spherical harmonic terms of order 2 or 4 could be detected, implying a minimum of 6 and 15 directions, respectively.

**Figure 4:**
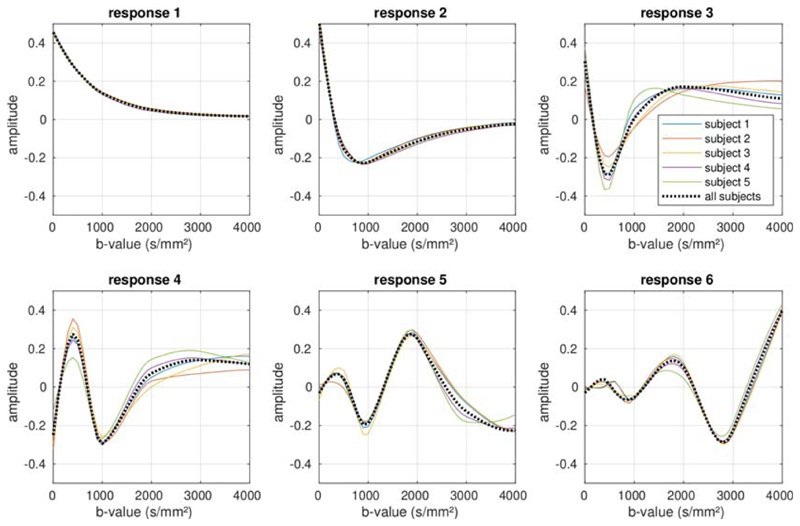
The basis functions observed in the data, for all five subjects included in the study. The basis functions estimated from each subject when analysed independently are shown by the solid coloured lines, and show a high degree of consistency across subjects. The dotted black line shows the basis functions estimated from the combined data across all subjects. The basis functions can be seen to represent increasingly rapid oscillations in the *b*-value dependence, with the first component corresponding to the mean signal across the data. See Table 2 for the corresponding effect sizes

**Figure 5:**
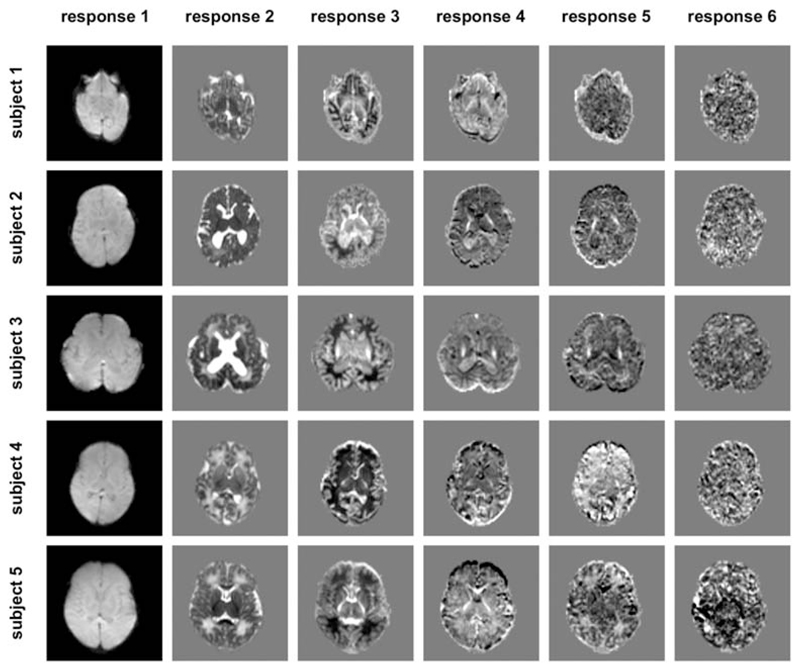
Weights maps for each component in Figure 4, for all five subjects included in the study

The basis functions *H* and weights maps *W* (cf. Equation 3) derived using SVD are shown in Figures 4 and 5, respectively, and are remarkably consistent across subjects. The first component corresponds to the mean DW signal over all voxels, with the higher-order components showing increasingly rapid oscillations with *b*-value. Clear structure can be observed in the corresponding weights maps for at least the first four components (although the fourth component may possibly contain some residual misregistration due to, for example, motion or eddy currents, as well as any actual biology-related structure). In our data, the fifth order lacks consistency across subjects, suggesting that the remaining spatial structure may be strongly related to artefacts in the data. The sixth and highest order that could be derived from the test data given the number of shells, lacks both consistency across subjects and anatomical coherence, suggesting that noise and artefacts have become completely dominant. The effect sizes for these components decrease rapidly for the higher-order coefficients, as shown in Table 2.

**Figure 6:**
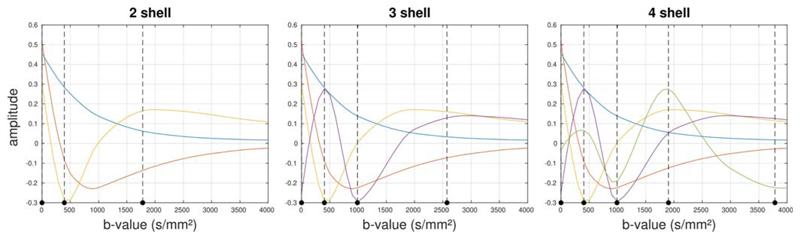
Optimal *b*-values selected by the algorithm, for 2-shell (left), 3-shell (middle) and 4-shell (right) cases, overlaid on the corresponding basis functions. The vertical dotted lines indicate the optimal *b*-values identified. Results obtained assuming a *T*_2_ value of 150 ms

**Figure 7:**
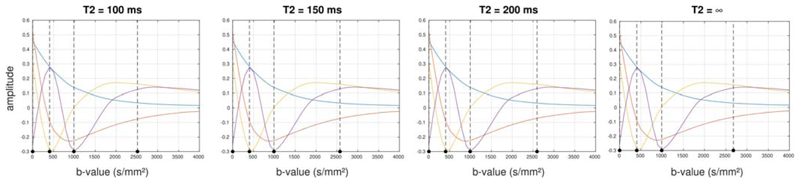
Effect of *T*_2_ on optimal *b*-values selected for the 3-shell case. The vertical dotted lines indicate the optimal *b*-values identified the *T*_2_ value stated for each plot. While *T*_2_ does have an influence, it is relatively minor, with lower *T*_2_ leading primarily to a slightly reduced maximum *b*-value, with little to no impact on the other selected *b*-values

The optimal *b*-values selected by the algorithm are shown in Figure 6 for the case of 2-, 3- and 4-shells (not including the *b*=*0* shell), assuming a *T*_2_ value of 150 ms in neonatal brain tissue.^53^ Figure 7 shows the effect of different *T*_2_ values for the 3-shell case. In all cases, a low *b*-value in the region of *b* = 400 s/mm^2^ is included, and can be seen to provide good separation between the first component and the other smaller components. In general, the algorithm seems to distribute *b*-values in a way that provides optimal separation between the different components, as desired. When more shells are included, the highest *b*-value selected increases, particularly when *T*_2_ relaxation is ignored. When relaxation effects are included, the maximum *b*-value never exceeds *b* ≈ 2600 s/mm^2^.

**Figure 8:**
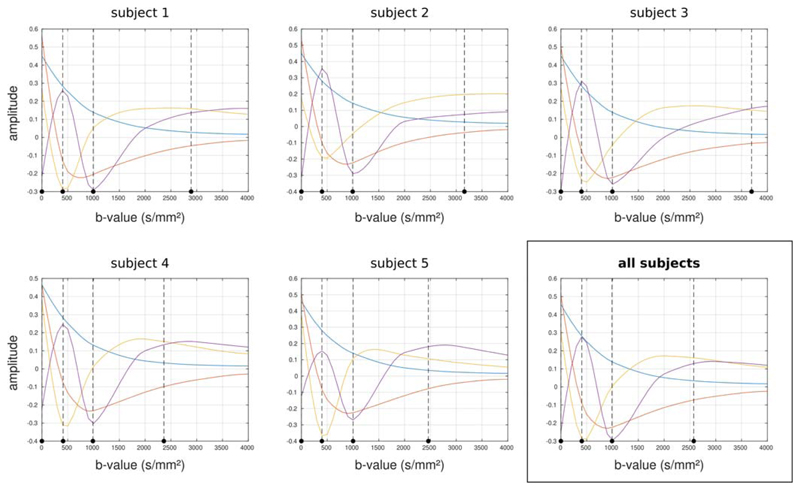
Optimal *b*-values selected by the algorithm when applied to each subject individually for the 3-shell case and assuming *T*_2_ = 150 ms. The vertical dotted lines indicate the optimal *b*-values identified in each case. While there is some variation in the maximum *b*-value, the other *b*-values remain very stable

The *b*-values selected as optimal are stable across subjects, apart from the maximum *b*-value, as shown in Figure 8 for the 3-shell case. The maximum *b*-value varies in this case from *b* ≈ 2300 to 3500 s/mm^2^; this is likely related to the signal levelling out as a function of *b*-value in this range, leading to a broad optimum.

Table 3 shows the optimal results for 2-, 3- and 4-shells assuming *T*_2_ = 150 ms (suitable for neonates at 3T^53^). Along with the optimal *b*-values, the optimal number of directions per shell are also listed as a percentage of the total number of volumes acquired. For reference, the CNR for the coefficients themselves is shown in Table 4, assuming SNR = 30 and *N_total_* = 100 imaging volumes. As expected, the CNR drops rapidly for the smaller coefficients, and is typically improved by using the smallest number of shells needed to characterise that coefficient (eg, *b=0* + 2 shells to characterise the third coefficient). These CNR calculations can be generalised to arbitrary conditions by adjusting them based on the actual number of volumes and SNR_*b=0*_ values:

(15) $${\text{CNR}}_{\text{actual}}={\text{CNR}}_{\text{listed}}\times \frac{{\text{SNR}}_{b=0}}{{\text{SNR}}_{\text{listed}}}\times \sqrt{\frac{N_{\text{total}}}{100}}$$

As expected, larger numbers of volumes are needed for the higher *b*-value shells. This is expected given the lower SNR available at these *b*-values, and also helps to ensure that the minimum orientation sampling density requirements (identified earlier using the angular frequency analysis) are satisfied.^30^

**Figure 9:**
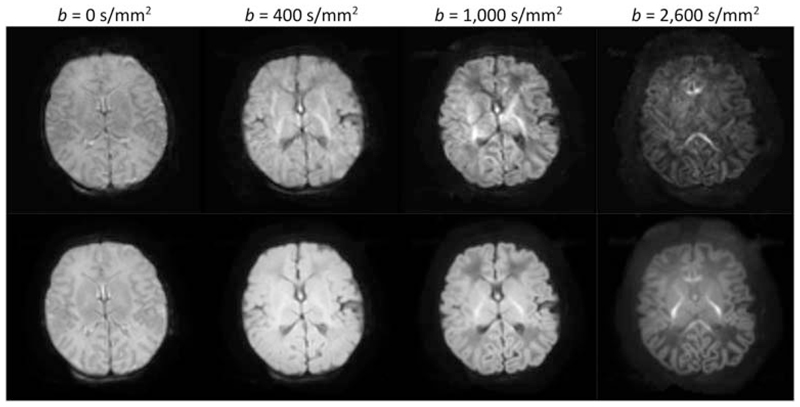
Single diffusion-weighted images (top row) and mean diffusion-weighted image (bottom row) for each shell for a typical subject acquired using the final dHCP protocol

Based on this optimisation, a 3-shell protocol was adopted for the dHCP.^54^ The acquisition includes a total of 300 samples distributed in the proportions listed in Table 3. Figure 9 shows example images and shell average images acquired as part of the dHCP study (PGSE EPI acquisition with multiband factor 4, 64 slices, TE/TR = 90/3800 ms, 1.5 × 1.5 × 3 mm resolution, four phase-encoding directions, SENSE 1.2, Partial Fourier 0.855), after correction for EPI distortion, subject motion and outliers.^38,39^ While the individual images in the highest shell display the expected low SNR, the shell average reveals a highly coherent anatomical structure confirming its information richness, which can be well accessed by virtue of the large number of samples collected. All shells have angular sampling in excess of the requirements determined from the pilot data.

## 4 Discussion

This study provides a data-driven framework to identify the optimal parameters for multi-shell HARDI acquisitions, based on an information-theoretic approach. It is independent of any particular reconstruction/diffusion analysis algorithm, which is a significant advantage given that many of these approaches are under active development, have a number of different tuneable parameters, and are designed to provide different types of information. As illustrated in the Appendix, a protocol optimised for any one of these would therefore not be guaranteed optimal for any of the other methods (or indeed, any other parameters or outputs of the same method). The proposed design method allows protocols to be implemented now that will remain future-proof as novel analysis approaches are developed.

While a number of decompositions could be used for the *b*-value dependence, singular value decomposition is arguably the most appropriate for this study. The SVD provides a unique and complete signal representation with a clear interpretation as components of decreasing effect size. In contrast to other factorisations that deploy biophysical constraints, for example, to enforce nonnegativity in both weights and basis functions,^55–57^ the SVD deliberately avoids any such constraints. As such, we can ensure that the optimised multi-shell HARDI scheme is driven by the data itself, and not by implicit assumptions of a particular tissue model.

The optimisation suggests that the optimal number of directions per shell can be expressed as a fraction of the total number of volumes to be acquired. Furthermore, the CNR of the estimated coefficients will scale with the square root of the total number of measurements. These results can therefore easily be tailored to the particular circumstances under which the protocol is to be used, using the recommendations outlined below:

1. Identify the desired imaging parameters (eg, resolution, spatial coverage) that will determine the SNR and repetition time of the sequence. Divide the scan time allocated to this sequence by theTR to obtain an estimate of the total number of volumes to be acquired.
2. Based on the SNR and total number of volumes, compute the CNR for the various coefficients for the 2-, 3- and 4-shell scenarios, using Equation 15.
3. Decide on the number of shells that will be used by selecting that which predicts usable CNR for all estimated coefficients (ie, the CNR is above the noise floor for all coefficients).
4. Using our previous orientation-domain approach,^30^ verify that the number of directions for each *b*-value is sufficient to avoid aliasing, given the noise level in the images.
5. If the number of directions per shell is not sufficient to avoid aliasing in the angular domain, the data acquisition will not provide optimal characterisation of the dMRI signal. There are several ways to address this issue:
  - Increase the number of volumes in the acquisition, and hence the scan time, to meet the minimum sampling requirements.
  - Select a lower number of shells, as this increases the number of directions for the remaining shells. This decision will depend on whether the error introduced by inadequate sampling of the *b*-value domain (i.e., having too few shells) is greater than that introduced by inadequate sampling of the angular domain (i.e., having too few directions).
  - Redistribute directions between shells to ensure the minimum sampling requirements are met, striving to maintain the number of directions per shell as close to those identified as optimal. While this implies a departure from the optimal parameters for overall CNR, the actual impact is likely to remain acceptable for small adjustments, and can be evaluated using the approach outlined in this study.

It is interesting to note that the *b*-values identified as optimal are very robust across subjects (Figure 8): for the 3-shell case, *b* = 400 and 1000 s/mm^2^ are identified in all subjects with high reproducibility (the range is *b* = 397-411 and 988-1001 s/mm^2^, respectively). There is more variation for the highest *b*-value (ranging from *b* = 2360 to 3700 s/mm^2^), as can be seen from Figure 8. Moreover, these values are remarkably consistent when different numbers of shells are selected (*b* = 400 s/mm^2^ is present in all schemes, *b* = 1000 s/mm^2^ is present for the 3- and 4-shell schemes) (Figure 6). However, given the smoothness of the principal components, minor variations in the exact *b*-values used are unlikely to have a large impact on the optimality of the acquisition.

Perhaps surprisingly, these values are also remarkably insensitive to changes in *T*_2_ (Figure 7). This can be explained by considering that to provide sensitivity to all *N_c_* components, acquisition schemes will need to include a range of *b*-values, so that the highest *b*-value will necessarily be high relative to the others. This places a constraint on the minimum achievable echo time: any reduction in the echo time (and hence the maximum *b*-value) entails a greater reduction in sensitivity than the increase in SNR. Furthermore, this relative insensitivity is also likely to be a consequence of the long *T*_2_ values observed in neonatal MRI at 3T, which typically range from more than 100 to 300 ms (see, for example, table 4 in Williams et al^53^). It is likely that the same analysis performed in adult data, where *T*_2_ values are considerably shorter, would show a stronger effect of *T*_2_.

**Figure 10:**
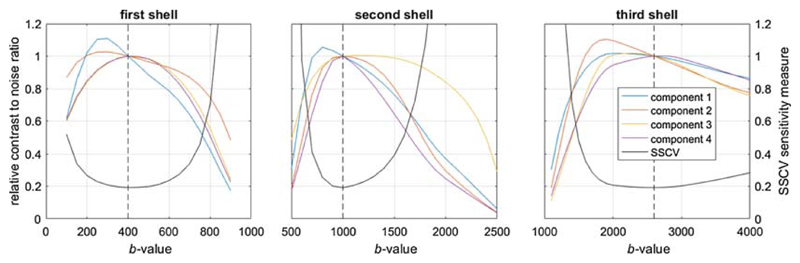
The impact of deviations from the optimal solution on the relative contrast-to-noise ratio (CNR) of each component (coloured lines) and on the sum of squared coefficients of variation (SSCV) measure (black line). Each plot shows the effect of changing the *b*-value of the corresponding shell away from the value identified as optimal (denoted by the dashed vertical line). The CNR for each component is shown as a fraction of its value at the overall optimum to allow all plots to coexist on the same scale. This suggests that the optimum is fairly broad, and performance is likely to be relatively tolerant of deviations in the *b*-values, particularly for the highest shell. In general, the acquisition is more sensitive to the largest component (component 1) with lower *b*-values, at the expense of sensitivity to the weakest components. This was produced assuming a scheme consisting of four *b*-value shells to estimate four components, and *T*_2_ = 150 ms

It is also interesting to look at the robustness of the acquisition to deviations in the parameters from the values identified as optimal. Figure 10 shows the effect of varying the *b*-value of each b≠0 shell in turn, in terms of its impact on the CNR of the individual components, and on the overall SSCV metric (the measure of sensitivity minimised in the present study). It is clear that higher sensitivity to individual components can be achieved, but this comes at the expense of sensitivity to other components. For example, lowering the first shell from *b* = 400 to *b* = 300 s/mm^2^ would increase sensitivity to the first component by ~10%, but would also reduce sensitivity to components 3 and 4 by ~10%. The SSCV measure shows a broad minimum, particularly for the highest shell, indicating that the acquisition would still perform well if the *b*-values differed somewhat from those identified.

Being entirely data-driven, the approach inherently provides parameters optimal for the particular cohort under investigation. In the present work, we focus on the neonatal age range, and the parameters identified should therefore only be considered optimal for this age range specifically. This approach could also be used to optimise protocols for ex vivo scanning, for instance. However, we note that the *b*-value optimisation procedure used here did not explicitly take the orientation information into account (beyond ensuring the minimum sampling density requirements are met); this approach may yield sub-optimal results if applied to the adult case where the angular dependence is much stronger. For instance, angular features in the signal have recently been shown to be critical to resolving degeneracies in the standard model^7^; since the approach taken here ignores these angular features, it cannot provide any guarantee of optimality for applications of this type. By contrast, the neonatal brain exhibits much lower anisotropy, and many of the most interesting features in this age range are likely to be better characterised by focusing on the *b*-value dependence of the mean DW signal (eg, Kaden et al^3^ and Reisert et al^32^), motivating in part the current approach. Joint optimisation of both angular and *b*-value dependences requires extended orthonormal *q*-space decompositions (*D*),^48^ and is the subject of ongoing work.

Due to its focus on the features of the raw signal, the optimisation performed here provides a solution that is generally suitable for any type of multi-shell dMRI analysis. However, it is likely that in certain applications, very specific aspects of the signal may be of interest, in which case a specially designed acquisition scheme may provide improved sensitivity for this particular purpose. We do, however, believe that such an acquisition would necessarily entail some loss of generality, with reduced sensitivity when used for other purposes.

As shown in Table 3, the lowest *b*-value selected by the algorithm, at b ≈ 400 s/mm^2^, is lower than has typically been used in previous multi-shell acquisitions (notably the Human Connectome Project), although it is in line with recommendations for diffusion kurtosis imaging (DKI)^1,58^ and neurite orientation density and dispersion imaging (NODDI).^6^ Furthermore, lower *b*-values are expected considering that mean diffusivity is higher in neonates than in the adult brain. On the other hand, the highest *b*-value selected is similar to those identified as optimal for fibre orientation estimation in adults^30,52^; the acquisition scheme should therefore be well suited to tractography applications. Finally, the number of DW directions per shell increases with higher *b*-value, most likely due to the stronger signal attenuation (coincidentally, increasing sampling density is also required to characterise the increasingly complex angular dependence of the signal at higher *b*-values).

A question that naturally arises from this work is whether and how the sampling directions should be optimised across shells. It has been suggested in previous work that the sampling directions should be chosen to provide uniform angular coverage across all shells.^59^ By contrast, in this study the directions were optimised for uniformity across each shell independently, with no attempt to introduce any interaction between shells. We motivate our approach using sampling theory, based on similar arguments to our previous work,^30^ as follows. We assume the diffusion signal is band-limited in the angular domain; that is, the diffusion signal varies smoothly as a function of orientation, and its angular frequency spectrum contains low harmonic terms only (depending on the *b*-value). By construction, our sampling scheme is sufficiently dense that all angular frequency components have been fully captured. When these conditions are met, there is no additional information to be gained by introducing inter-dependencies across shells, since the signal is already fully sampled. Furthermore, any attempt at introducing cross-shell dependencies can only push the sampling scheme away from per-shell uniformity, and hence compromise its overall quality. We therefore feel justified to optimise the directions on a purely per-shell basis (we do however note that optimisation across shells may be beneficial in the undersampled case, where each shell is not sufficiently sampled in isolation to capture all features of the signal; in this case, a suitable model of the signal may be able to make use of the complementary orientational information provided by the different shells).

Identifying the optimal number of shells to be included in the final protocol necessarily requires additional factors to be considered, most notably *N_total_*, which is in turn dependent on details of the acquisition and the total feasible acquisition time. The minimum angular sampling requirement imposes an upper limit on the number of shells that can be acquired for a chosen *N_total_* since increasing the number of shells necessarily reduces the number of directions per shell. However, these requirements are easily met for large *N_total_*, as is the case for the dHCP. It is notable that adding higher shells can strongly suppress the number of samples available for lower shells as, at least in the neonatal case, almost 50% of the samples must be obtained using the maximum *b*-value. We used the per-voxel maps of weights for each component to help inform this balance, based on an expectation that meaningful components should exhibit anatomically plausible spatial patterns that are consistent across subjects. As shown in Figure 5, the maps for the fourth component do exhibit some anatomically plausible contrast (notably in the posterior limb of the internal capsule), whereas the fifth component is much less consistent across the test subjects, suggesting it was strongly impacted by noise and artefacts, probably related to residual misregistration. For this reason, and given the nonnegligible drop in CNR for all components when increasing the number of shells (Table 4), we decided to use a 3-shell (+ *b*=*0*) scheme for the final dHCP protocol, with 300 volumes acquired within 20 minutes using multiband factor 4.^54^

## 5 Conclusion

The approach presented in this study provides a means to design an optimal acquisition multi-shell HARDI protocol, based on maximising the information content of the data, independent of any particular reconstruction method. It is based on empirically measured data, with no reliance on any assumed model of microstructure. It focuses primarily on the mean DW signal per shell, with separately derived safeguards to ensure adequate angular sampling; this is appropriate for neonatal data given the low angular contrast in this age range. The analysis performed here suggests that a *b*=*0* + 3 shells acquisition is suitable for neonatal imaging, consisting of *b* = 0, 400, 1000 and 2600 s/mm^2^ with numbers of DW directions per shell in proportions of 6:19:28:47; these were employed in the final diffusion MRI protocol for the dHCP project.

## Supplementary Material

Figure S1

Appendix

## Figures and Tables

**Table 1:** Details of the subjects included in this study

GA (weeks)	PMA (weeks)	Clinical features	Imaging findings
39 + 2	45 + 4	Smith-Lemli-Opitz syndrome	Cerebellar hypoplasia, small hypothalamic hamartoma
38 + 0	40 + 1	Fetal alcohol syndrome	Small but otherwise normal imaging appearance
40 + 5	42 + 5	Seizures, antenatal ventriculomegaly	Perisylvian polymicrogyria
33 + 3	36 + 1	Preterm	small germinal matrix haemorrhage
39 + 6	40 + 5	Meconium aspiration, mild hypoxia	Normal imaging appearance

**Table 2:** Effect sizes for each observed component in the decomposition (as shown in Figure 4, across all subjects (arbitrary units)

component	1	2	3	4	5	6
**effect size**	1.000	0.134	0.038	0.018	0.009	0.004

**Table 3:** Optimal *b*-values (in s/mm^2^) for each shell and corresponding number of directions (*N_total_*), expressed as a percentage of the total number of volumes acquired. Results are shown for the 2-shell, 3-shell and 4-shell cases, assuming a *T*_2_ value of 150 ms


2 shells	*b*-value	0	395	1781		
	*N_total_*	12%	29%	59%		

3 shells	*b*-value	0	404	990	2574	
	*N_total_*	6%	19%	28%	47%	

4 shells	*b*-value	0	405	990	1901	3784
	*N_total_*	3%	9%	16%	29%	43%


**Table 4:** Contrast-to-noise ratios (CNR) for each component, as would be estimated using the corresponding parameters in Table 3. These values assume a *T*_2_ value of 150 ms, a total of *N_total_* = 100, and an SNR in the *b*=*0* images of 30

component	1	2	3	4	5
2 shells	152	18	6.0		
3 shells	125	16	4.5	2.7	
4 shells	88	13	4.0	2.1	1.3

## Highlights

- A data-driven method is presented to design multi-shell diffusion MRI acquisition schemes (*b*-values and no. of directions).
- This method optimises the multi-shell scheme for maximum sensitivity to the information content in the signal.
- When applied in neonates, the data suggest that a *b*=*0* + 3 shell strategy is appropriate.

## Abbreviations used

CNR: contrast-to-noise ratio
CV: coefficient of variation
dHCP: developing Human Connectome Project
dMRI: diffusion magnetic resonance imaging
DTI: diffusion tensor imaging
DKI: diffusion kurtosis imaging
DW: diffusion-weighted
EPI: echo planar imaging
FA: fractional anisotropy
HARDI: high angular resolution diffusion imaging
IWLS: iteratively reweighted least-squares
MD: mean diffusivity
NODDI: neurite orientation density and dispersion imaging
OLS: ordinary least-squares
PGSE: pulsed gradient spin echo
SENSE: sensitivity encoding
SNR: signal-to-noise ratio
SSCV: sum of squared coefficients of variation
SVD: singular value decomposition
TE: echo time
TR: repetition time
WLS: weighted least-squares
