# Supplementary material for: A data-driven approach to optimising the encoding for multi-shell diffusion MRI with application to neonatal imaging
Source: NMR Biomed. Author manuscript; Available in PMC 2020 Nov 24. (PMC7116416; doi:10.1002/nbm.4348)
Supplement: Figure S1 [file EMS88518-supplement-Figure_S1.pdf]

Supplementary Material

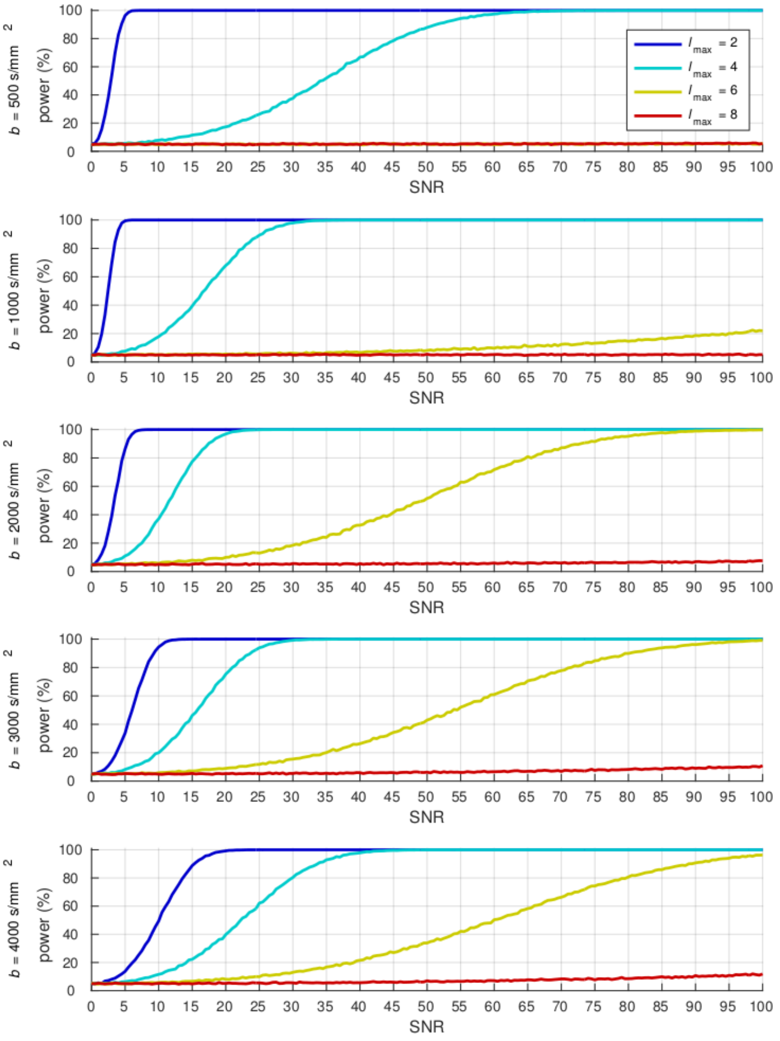

Supplementary Figure 1: The power to detect each axial spherical harmonic (SH) coefficient plotted as a function of the signal-to-noise ratio (SNR) in the *b*=0 image and assuming a clinically realistic scheme with N_{DW}=100, for each *b*-value shell independently. Each line corresponds to the power to detect a significant improvement (p<0.05) to the fit by including the corresponding l_{max} SH terms. This plot was generated using the same procedure as Figure 5 in (Tournier et al., 2013), which corresponds to the adult case.
