# Supplementary material for: A data-driven approach to optimising the encoding for multi-shell diffusion MRI with application to neonatal imaging
Source: NMR Biomed. Author manuscript; Available in PMC 2020 Nov 24. (PMC7116416; doi:10.1002/nbm.4348)
Supplement: Appendix [file EMS88518-supplement-Appendix.pdf]

APPENDIX

A.1 | Optimising against DTI indices

To illustrate the issues inherent in using specific model parameters to optimise general acquisition parameters, we performed a simple analysis based on the diffusion tensor model, geared towards identifying the optimal subset of *b*-value shells from the full available set, an approach similar to that used in a recent study.[60] This analysis relies on the availability of an oversampled acquisition that can serve as the "reference", and from which candidate acquisitions can be formed by taking subsets of the data. It is premised on the notion that the best reference reconstruction is obtained by taking all available data into account. To be deemed optimal, a candidate acquisition should provide results that closely match these reference results. Here, we investigate all possible 2-shell subsets of the reference data (all $b=0$ volumes were included in all candidates), as listed in Figure 1. All candidate schemes therefore consist of the same amount of data: five $b=0$ volumes + 2×50 DW volumes.

The data collected as part of this study provide an oversampled acquisition suitable for this type of analysis. To explore the issues with such an approach, we tested three different commonly used diffusion tensor fitting algorithms: an ordinary least-squares fit to the log-signal (OLS); a weighted least-squares fit to the log-signal (WLS); and an iteratively reweighted least-squares fit to the log-signal (IWLS).[26–29] For each fitting approach, the reference reconstruction and all candidate reconstructions were computed using that approach, and the results of each candidate acquisition were always compared only with the reference computed using the same fitting algorithm.

As in Howell et al,[60] we look at FA and MD as the outcome metrics of interest, and quantify the discrepancy between each candidate acquisition and the reference by computing the absolute difference in the computed metrics, normalised to the corresponding reference value. The median absolute difference is computed within a conservative brain mask for each subject, and subsequently averaged across subjects. The resulting discrepancy values are shown in Figure 1, for FA and MD (columns) and all three fitting algorithms (rows), for all candidate diffusion

schemes (x-axis). The OLS results differ markedly from the other fitting approaches, with more minor differences between WLS and IWLS. More importantly, the acquisition identified as "optimal" differs depending on which fitting approach and output metric was used.

In addition, we also computed the discrepancy between the reference results computed using the different fitting approaches. For any two of the fitting approaches, the absolute difference in the reference values produced (i.e., obtained using all available data) were computed, and the median calculated. These results are shown in the bottom right panel of Figure 1, for both FA and MD metrics, for all pairs of fitting methods. Note that the differences observed in this case are as great or larger than those observed in the previous analysis. In other words, the estimator used to compute the metrics has a greater impact on the results than the combination of b-values used in the acquisition. Furthermore, in the absence of ground truth, there is no principled way to decide which fitting algorithm should be used.

As can readily be appreciated, there is little consistency in these results, making it near-impossible to identify which of these schemes can legitimately be claimed to be optimal in any sense. Furthermore, it should be noted that the diffusion tensor is unlikely to be appropriate particularly at high b-values; the results shown here are likely to reflect deficiencies in the model, rather than genuine differences in performance. These results would no doubt differ again if other higher-order models had been included in the comparison, including, but not limited to: Q-ball imaging,[61–63] ball and sticks,[64] NODDI[6] and/or spherical deconvolution.[2,55,65] Moreover, most of these models can also be fitted using different approaches, each with different user-adjustable parameters (eg, amount of regularisation, harmonic order), and produce different types of output metrics. With such a wide range of models, parameters and outcomes, it is clear that a different approach is required, motivating the current study.
